# More Information, Greater Appreciation: The Correlation between Background Information and Aesthetic Judgment of Tourist Crafts

**DOI:** 10.3390/bs12070217

**Published:** 2022-06-29

**Authors:** Yang Liu, Jie Zhang, Shiwei Shen, Kaixiang Lu

**Affiliations:** 1 Joint Institute of Ningbo University and University of Angers at Ningbo, Ningbo University, Ningbo 315211, China; amyliuyang0818@foxmail.com (Y.L.); jiezhang@nju.edu.cn (J.Z.); shenshiwei@nbu.edu.cn (S.S.); 2 ESTHUA, Faculty of Tourism, Culture and Hospitality, University of Angers, 49004 Angers, France; 3 School of Geography and Ocean Science, Nanjing University, Nanjing 210023, China

**Keywords:** art psychology, behavioral psychology, tourism aesthetics, aesthetic judgment, background information, art interest, intangible cultural heritage, Chinese tourist crafts, blue calico, Chinese tourists

## Abstract

More information is often correlated with greater appreciation. Drawing on the model of aesthetic appreciation and aesthetic judgment in art psychology, this study aims to investigate changes in tourists’ aesthetic judgments of tourist crafts when provided with different background information. Blue calico, an art form created through white pulp dyeing and printing, is an intangible cultural heritage of China. The photographs used in this study illustrate typical examples of blue calicos that are commonly sold in tourist gift shops in Wuzhen, China. Data from a sample of 133 participants (49 women and 84 men) was analyzed using Two-Way Repeated Measures ANOVA. We examined to what extent respondents varied their assessments of the calicos based on author manipulation of background factors, such as commentaries by the artist or details about the production process. We found that tourists’ impressions of the aesthetics of blue calicos were predicted by background factors, especially those of tourists who were less interested in high arts. Specifically, blue calicos reported to tourists with names that conveyed an auspicious meaning predicted tourists’ assessments of the calicos as more aesthetically pleasing. Explanations of the production process also predicted an increased appreciation of calico aesthetics. Conversely, artists’ commentaries were not significantly correlated with an increased aesthetic merit of calicos. Understanding what may affect tourists’ assessment of art could help those in the tourism industry market souvenirs to drive sales and enhance tourists’ understanding and appreciation of intangible cultural heritage.

## 1. Introduction and Literature

Crafts are a standard element at tourist destinations and have long played an important role in the tourism industry [1]. Evans provided a detailed definition of the term crafts in 1994 as “a type of work where useful and/or decorative devices are made by hand or with simple tools” [2]. For the modern tourism industry, however, this may not fully apply as the scale and nature of tourist demand exceeds the capabilities of the hand-made level [1]. With the development of tourism, artisans are not solely limited to local needs, but are beginning to fulfill the needs of other regions. They began to transform their handicrafts from practical objects to tourist crafts, based largely on tourists’ expectations of what a souvenir should be [3]. During this process, the form, function, meaning, and symbolism of the crafts changed [4]. Tourist crafts, developed and innovated based on the original crafts, are the new products that accompany the development of modern tourism. Produced and developed mainly to meet the needs of the tourism market, they are often described as “authentic replicas” [2], “tourist art” [5], or “airport art” [6], indicating a mass-produced type of manufacturing. Still, even if they are fully or partially mass-produced, they may, as objects, reflect or imitate traditional materials, techniques, and a sense of uniqueness.

Blue calico is a folk handicraft with the characteristics of Jiangnan (simple and elegant) produced using the traditional Chinese process of creating blue and white traditional prints on cloth using pattern templates and dye created from natural pigments [7]. With a history of over 1300 years, it has been included in the first group (Item No.Ⅷ-24) of the “National Intangible Cultural Heritage List [8].” and is a valuable traditional dyeing technique in China, in addition to Miao batik, Li Ikat dyeing, and tie-dyeing. As a specific commodity of intangible cultural heritage (ICH), blue calico plays an important role in its protection and transmission. Attributed as one of the original locations of blue calico production, Wuzhen has a long history of maintaining a unique cultural connotation with the craft, making it a fascinating tradition. With the development of tourism, the blue calico of Wuzhen has transformed into a successful tourist craft, which tourists can often view in the attractions. Thus, we chose blue calico of Wuzhen as the stimuli for our experiments.

Aesthetics is the study of sensory or sensory–emotional values [9]. Tourism and aesthetics are so closely integrated that Ackerman [10] argues that one cannot exist without the other. Tourism aesthetics is multidisciplinary, spanning several fields, including experimental aesthetics and tourism experience and tourism culture [11]. In the book *Chinese Tourism Literature*, Mr. Feng Naikang argues that tourism “is a short-term way of life in which one seeks aesthetic enjoyment in a foreign place”. In fact, tourism aesthetics is very important because it deals with the ontological properties of tourism. Although the term aesthetics is frequently used in the tourism literature [12,13,14,15], the topic is seldom fully explored [9]. As evidenced by the scant number of papers, recent research on tourism aesthetics has been relatively weak in relation to other tourism research and has focused on the ecological service value of tourism [16] and its cultural ecosystem service value [17]. Existing tourism aesthetic studies focus primarily on natural landscape aesthetics [18], music [19], photographs [20], Chinese gardens [21], sculptures [22], calligraphy art [23], architecture [24], food [25,26], and the tourism experience [27], while tourism crafts are not considered. Regarding the subject of tourism aesthetics, it mainly studies aesthetic education [28], aesthetic preference [29], aesthetic appreciation [30], and aesthetic experience [31], and it incorporates the content of aesthetic emotion [32] and psychological consciousness of aesthetics from the tourists’ perspective [33]. Tourists’ liking can be correlated with exposure to an aesthetically pleasing environment with constant stimuli that create a positive attitude toward the environment [34]. The research methodology is mainly qualitative and lacks quantitative analysis [35]. This study also attempts to enrich the theoretical nature of tourism aesthetics by citing the theory of art psychology. The study of tourism aesthetics in China began in the mid-1980s. The results of Western aesthetics research have always greatly influenced the research of Chinese scholars [36]. However, the development of tourism in China has its own peculiarities [37]. The symbiosis of agrarian, industrial, and information societies and the significant east–west, urban–rural, and population quality differences have led to differences in tourism demand and aesthetic needs [36]. Therefore, by analyzing the otherness attributes of Chinese tourists, it is clear that localized tourism aesthetics research is the future trend of tourism aesthetics development in China. As a collection of cultural experience, cultural creativity, and local art, the aesthetic experience and the aesthetic judgment of tourism crafts are important and typical elements of tourism aesthetics research.

Leder et al. [38] conceptualized aesthetic judgment as “a result of the Evaluation of the Cognitive Mastering stage” in the model of aesthetic appreciation and aesthetic judgments [18]. When people see an object, they can easily judge its beauty; however, it is difficult to answer the question of how humans make such aesthetic judgments. Identifying the factors that correlate with aesthetic judgments can answer this question [39]. To analyze subjects’ aesthetic judgments of artworks, several predictor variables (variables that come earlier in an explanation) were verified. As many studies have demonstrated, symmetry, complexity, or combinations of objects correlate with viewers’ aesthetic judgments [40,41,42]. Another factor that correlates with aesthetic judgments is the knowledge about the object, which can determine our aesthetic value [30]. Aesthetic judgment is not only largely subjective, but also highly dependent on the cultural background of an appreciator [43]. Existing research has demonstrated that content, such as title and background information, predict people’s appreciation of abstract paintings, including meaningfulness and pleasure value [30,39,44]. In terms of the aesthetic experience of a vast majority of the viewers, the provision of titles and moderate descriptive text positively correlates with the aesthetic pleasure and meaningfulness of the works presented in the exhibition, regardless of whether they are figurative or abstract paintings. The experimental results indicate that the provision of appropriate text, such as titles, artist information, and background descriptions of artworks, can indeed help viewers better understand the works, stimulate aesthetic pleasure, and optimize the aesthetic experience. In addition, the importance of interest in key concepts influencing artistic judgments is also reflected in current theories and models of aesthetic appreciation [38,45]. Interest in art and knowledge of art are arguably the central dimensions of art experience and the two most important individual differences in assessing how people process or respond to art [46]. Interest in the arts was measured as an interpersonal difference to confirm that increased interest facilitates higher comprehension and to verify whether interest in the arts interacts with any other variables. However, to date, most studies in the field of aesthetics have focused on high arts. With the aestheticization of everyday life [47] and the evolution of tourism as a common activity, its aesthetic study has become more and more important. It is still not known whether differences exist between the aesthetic study of tourism and the aesthetic study of high art. Thus, a gap exists in the literature related to tourism research and whether aesthetic judgments of tourist crafts are correlated with these predictor variables.

In addition to the study of aesthetic influences, the role and impact of aesthetics has also been validated in research. Empirical evidence in various service environments has demonstrated that customer experience will inevitably be affected by surrounding aesthetic cues, which are the aesthetic elements in the surrounding environment. For example, studies have found that facility aesthetics correlate with perceived servicescape quality, which in turn affects satisfaction, intention to revisit, and desire to stay [48]. Aesthetic dining environments can affect perceived food and service quality and directly correlate with behavioral intentions [49]. At the same time, aesthetic judgments play an equally important role in the assessment of the overall experience [50], and may or may not lead to the occurrence of aesthetic pleasure. Finally, the effect of the aesthetic perception of goods on usability has been well studied, resulting in fairly consistent conclusions. More attractive goods are also perceived to be easier to use [51]. The positive effects of aesthetics have been validated in a variety of national cultures, including Japan [52], Israel [51], Switzerland [53], Germany [54], and the UK [55]. In addition, a range of goods, including cell phones [56], ATMs [51], web pages [55,57,58], and video games [54], have all been validated for their aesthetic importance.

Based on the analysis and review of such literature, we determined that the aesthetic study of tourism crafts has not been given much attention in tourism aesthetics and a research gap exists. In addition, differences in the influencing factors for the study of tourism aesthetics and high art aesthetics remain undetermined. Existing research recognizes the critical role that aesthetics play; therefore, studying the aesthetics of tourism crafts is very meaningful. In addition, our research draws on the models in aesthetic psychology to extend the study of aesthetics to the field of tourism. Further, a relatively complete theoretical system in the basic theoretical research of aesthetic psychology exists [59], which provides a solid theoretical foundation, research ideas, and research methods for this study. Thus, this study is feasible. Based on the above analysis, our main research questions are as follows:

1. Is there heterogeneity in the aesthetic judgment of tourist crafts? What individual differences exist?
2. Are tourists’ aesthetic judgments of tourist crafts predicted by background information?

Based on the goals of our research, we proposed the following hypotheses:

**Hypothesis** **1.** *Background information is positively related to the aesthetic judgment of tourist crafts.*

**Hypothesis** **2.** *Art interest is positively related to the aesthetic judgments of tourism crafts.*

**Hypothesis** **3.** *The aesthetic judgment of tourists with high and low interest in art is related to background information to different degrees.*

This study adopts a self-report questionnaire of experimental aesthetics and a model of aesthetic appreciation and aesthetic judgment in aesthetic psychology, and it focuses on blue calico of Wuzhen as the object to empirically test the correlation between background information and aesthetic judgment of tourism crafts. The study aims to enrich research related to tourism aesthetics and art psychology at the theoretical level. At the practical level, it intends to innovate and optimize the packaging and display design of tourism crafts to satisfy tourists’ requirements for higher aesthetics [60], and thus to promote the development of the tourism craft industry. At a macro level, we hope to enhance tourists’ understanding and appreciation of intangible cultural heritage by studying the aesthetics of tourism crafts.

## 2. Materials and Methods

### 2.1. Participants

The subjects were selected to participate in the experiment through random sampling in the Wuzhen West Scenic Zone, one of the sightseeing areas in Wuzhen. We regarded people who were taking photos or resting with their backpacks as tourists. We conducted a pre-study from 22 to 24 June 2020, which is a small-scale experiment conducted before the formal research. The sample comprised 16 experimental participants, including 8 men and 8 women. In the pre-study, we tested the experimental procedure. Some unclear words in the self-report questionnaire were modified based on the participants’ feedback. In general, our experiment went well and the self-report questionnaire was well designed.

Based on the calculation in Cohen [61] and related research [62], the sample size was determined by means of a priori power analysis, performed using G*Power3.1, to test repeated ANOVA measures, with an alpha of 0.05 to achieve a power of 0.80. The planned sample size included more than 86 people. Therefore, formal research was conducted between 11–20 February 2021. Researchers collected 145 questionnaires, 12 of which were incorrectly completed, and thus deleted; the final sample consisted of 133 participants (49 women and 84 men). All participants signed an informed consent document.

Table 1 presents the demographic profiles based on those who provided the richer datasets in post exhibition questioning. More than half of the participants (72.2%) have a bachelor’s degree or above, and 63.2% were students. Just under half (49.6%) received a monthly income of under 2000 RMB.

The correlation analysis between sociodemographic information, art interest, and aesthetic judgment showed that there is no significant difference among them (Table 2).

### 2.2. Stimuli

In the Wuzhen Scenic Area, we observed that the names of the products in the “Blue Calico” store are very brief, with only “square towel” and “handkerchief”, as well as information on dimensions. The names of the patterns on the blue calicos are not introduced. In addition, the title “Intangible Cultural Heritage” and celebrity evaluations of the blue calico are not shown. These phenomena raise the question as to whether tourists’ aesthetic judgments are correlated with background information, such as titles, the production process, and cultural meaning. Therefore, our study uses blue calico, a traditional Chinese handicraft, as the experimental material to investigate the correlation between background information and tourists’ aesthetic judgments.

**Figure 1:**
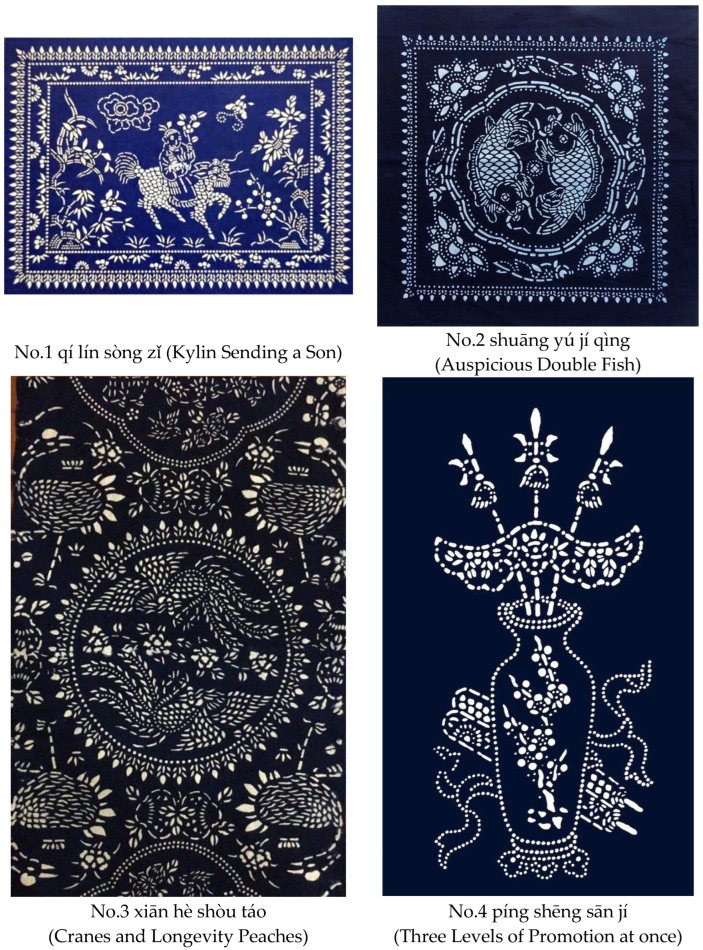
Photographs of the four blue calicos and their names.

We showed photographs and videos to participants on an iPad as the stimuli of the experiment. The four photographs of blue calicos shown to participants were provided by the owner of a blue calico store (see Figure 1). The first one is titled qí lín sòng zǐ, which means Kylin sending a son. The second one is titled shuāng yú jí qìng, which means auspicious double fish. The third one is titled xiān hè shòu táo, which means cranes and longevity peaches. The fourth one is titled píng shēng sān jí, which means three levels of promotion at once.

Three video clips of information required for the experiment—the product name, artist’s comments, and the production process—were obtained from the official website [63,64]. The name of each blue calico is introduced in the first video. Mr. Wu Yuanxin, a master of Chinese arts and crafts, comments on blue calico in the second video. The process of making blue calico is shown in the third video.

### 2.3. Procedure

Because we wanted to include tourists who were visiting Wuzhen as participants, we chose the method of field experiment to better reach our target subjects. Although field experiments have the disadvantage of the difficulty of controlling experimental variables compared to laboratory experiments, they have the advantage of being incomparable to laboratory experiments, such as the authenticity of the subject sample and the unique properties of the experimental site. After comprehensive consideration, we chose to conduct the experiment in the boardroom of the Muxin Art Museum in the Wuzhen West Scenic Zone. It is a very quiet place with minimal people. Participants participate in experiments one by one in a separate space away from other participants who might influence their answers. We wanted to ensure that the experimental environment was undisturbed and to control the variables as much as possible.

**Figure 2:**
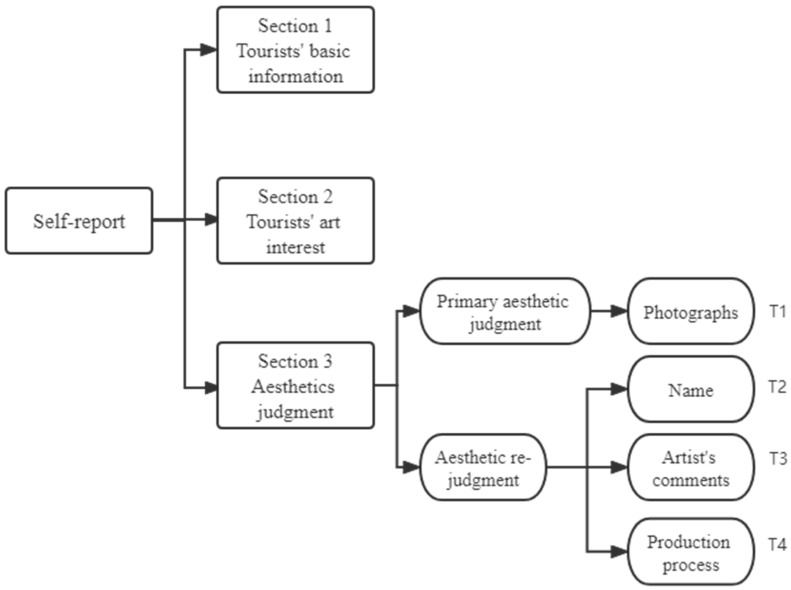
Flowchart of the experiment.

Participants were asked to report the degree of their aesthetic judgment for each stage through a self-report questionnaire with no time limit. In the first part, participants’ basic information was completed. In the second part, an art interest questionnaire [46] was administered to participants to distinguish the degree of individual art interest. In the third part, participants were asked to make their first aesthetic judgment after freely viewing four photographs of blue calico (T1), to make an aesthetic re-judgment after watching a video with the names of each of the blue calicos (T2), to make an aesthetic re-judgment after watching an artist’s evaluation of blue calico performing aesthetic re-judgment (T3), and after watching a video of the production process of blue calico (T4). Existing studies show that a presentation time of 10 s is sufficient for the aesthetic of a painting [44]. Therefore, this experiment allowed the tourists to freely view the images without the effect of time on aesthetic judgments, such as comprehension. Figure 2 illustrates a detailed flowchart of the experiment.

### 2.4. Self-Report Questionnaire

The Vienna Art Interest and Art Knowledge (VAIAK) questionnaire is a validated instrument for measuring interest and knowledge of art. It consists of two subscales, art interest and art knowledge. Because the art knowledge scale requires participants to answer art-related factual knowledge, which is difficult for tourists, it is not suitable for use in our study. Therefore, we only used the art interest subscale of VAIAK to measure tourists’ interest in art [46]. The art interest scale comprises two components—art interest and frequency of artistic behavior—with 11 total question items. To make the scale more applicable to tourism research, we added three new items for measuring art interest as follows: “During the tour, I like to visit artworks and art exhibitions”, “During the tour, I like to participate in art creation activities”, and “During the tour, artistry is an important reason for my travel considerations”. We modified the expressions in terms of individual words and statements according to the tourism context. For example, we added “during the tour” in front of the item “I am always looking for new artistic impressions and experiences”. The final questionnaire for art interest consisted of 14 items. The specific items used in the questionnaire can be seen in Table 3. We invited three professors in the field of tourism to review our new items, all of whom gave positive responses. VAIAK is a scale in English, and no Chinese research has used this scale to date. Thus, we invited two post-graduate students from the English department to translate the scale into Chinese.

The components of aesthetic judgment used the six dimensions from Leder et al.’s [44] study: understanding, meaning, liking, interest, emotion, and thoughts. We used the specific tourist craft of blue calico as the object in all dimensions. The detailed content is as follows: (a) understanding was measured using a scale that determined whether the participants believed they understood the intention of the blue calicos; (b) meaning, by whether they found personal meaning in the blue calicos; (c) liking, by whether they liked the blue calicos; (d) interest, by whether the blue calicos evoked their interest; (e) emotion, by whether the blue calicos affected them emotionally; and (f) thoughts, by whether the blue calicos evoked thoughts in them. We used a seven-point Likert scale from one (fully agree) to seven (fully disagree) to measure the items.

The scale was analyzed for reliability and validity using SPSS 25.0. As dimension reduction techniques seek to identify items with a shared variance, it is advisable to remove any item with a communality score less than 0.2 [65]. The results showed that the communalities >0.2 [65] and factors loading >0.4 [66] of all items were acceptable. The internal consistency of instrument subscales was measured using their Cronbach’s alpha coefficient (*n* = 20), where the least Cronbach’s alpha ≥0.650 was considered as acceptable [67]. The Cronbach’s alpha of the scale was 0.980, so the overall reliability of the questionnaire was high. Therefore, all items of the questionnaire were initially retained.

Prior to the extraction of the factors, several tests should be used to assess the suitability of the respondent data for factor analysis. These tests include Kaiser–Meyer–Olkin (KMO) Measure of Sampling Adequacy and Bartlett’s Test of Sphericity [68]. KMO was applied to determine sampling adequacy for factor analysis [65]. With a KMO of 0.930, the current sample was determined to be “very applicable to factor analysis” [69]. The significant Bartlett’s Test of Sphericity (χ^2^(190) = 2591.1, *p* < 0.001) indicated that the scale was suitable for factor analysis [68].

The validity of the questionnaire was then verified by exploratory factor analysis (EFA); the results of which are detailed in Table 3. The data were analyzed by exploratory factor analysis using principal component analysis and Kaiser’s normalized maximum variance method. One of the most popular criteria used for determining the number structures is the Kaiser Criterion or eigenvalues >1, which retains factors with eigenvalues >1 [65]. The scree plot showed a total of three factors with eigenvalue >1, with the three factors together able to explain 75.526% of the overall variance. If it is greater than 60% [70], these three factors can be extracted.

### 2.5. Data Analysis

Statistical analyses were performed using Statistical Package for Social Science (SPSS; version 25.0; Armonk, NY, USA) and the significance level was set at 5%. Descriptive statistics were acquired for gender, age, and years of education. The data analysis methods in the first part of this study were descriptive statistics and ANOVA. In the second part, repeated-measures ANOVA was conducted, with “interest in art” as a between-subjects factor (divided into a high art interest group and a low art interest group by cluster analysis), “background information” as a within-subjects factor (T1, T2, T3, T4), and aesthetic judgment as a measurement factor. Repeated measures ANOVA was conducted. The third part of the analysis was conducted to analyze the interaction between background information and interest in art.

## 3. Results

### 3.1. Descriptive Analysis for Men and Women

All descriptive statistics for men and women are presented in Table 4. Since all *p* values are greater than 0.05, it means that there is no significant difference between men and women in terms of art interest and aesthetic judgment.

### 3.2. Regression Analysis

Firstly, we examine the distribution of data and residual errors. For the data of art interest, the sig value = 0.443; it is greater than 0.05 (normally distributed data). The Normal P-P Plot of Regression Standardized Residual shows that residual errors are normally distributed. Secondly, we regressed the mean scores of all tourists’ art interest questionnaires on the scores of tourists’ primary aesthetic judgments (Table 5).

Y = −0.526 + 0.027X + e

Y = Aesthetic judgment

X = Art interest

The explanation of the regression equation above is as follows: a constant of −0.526 means that if the variable of art interest is not included in the study, the aesthetics judgment of tourists will decrease by 0.526%; art interest regression coefficient is 0.027, which means that if the art interest increases, it will increase aesthetic judgment. So, any increase in art interest will increase aesthetic judgment.

Finally, we assess the feasibility of the regression model. In view of the results of the analysis according to Table 5, there is a significant proportion of variance in aesthetic judgment, R^2^ = 0.740, F (1, 131) = 372.261, *p* < 0.001. The value of R^2^ shows that 74% of the aesthetic judgment variable is explained by art interest, which means 26% of the aesthetic judgement is explained by other variables not mentioned in this study. The F test is used to test the goodness of fit or the feasibility of the regression model, whether the model used in the research is fit or not. The calculated F value is 19.294 while the F table value is −2.206, and thus the F counted > F table. So, this model has a well goodness-of-fit. The value of *p* shows that art interest significantly influences aesthetic judgment. Thus, hypothesis 2 is supported.

### 3.3. Cluster Analysis

In order to investigate whether there are differences between tourists with different art interests influenced by background information and whether there is an interaction effect between art interests and background information, we conducted a cluster analysis of tourists. In addition, grouping tourists can also help enlighten tourist craft shops in practical applications. Cluster analysis is the task of grouping a set of objects in such a way that objects in the same group (called a cluster) are more similar (in some sense) to each other than to those in other groups (clusters). Based on the score of art interest questionnaire, we used a k-means method of quick cluster in SPSS to divide the 133 participants into a low art interest group and a high art interest group. The high art interest group (Cluster 1) included 51 participants and the low art interest group (Cluster 2) contained 82. The results of the cluster analysis are presented in Table 6.

### 3.4. Pairwise Comparisons

Table 7 presents the mean and standard deviations of the participants’ aesthetic judgments between the two groups at different time points. The correlation between each piece of information and tourists’ aesthetic judgment was assessed by the increase in the six factors, which was a comparison of the judgments made before and after exposure to each piece of information. Repeated measures ANOVA was used to verify the level of significance of the information effects. Aesthetic judgments of blue calicos correlated with each piece of information were compared between two groups of participants with different levels of art interest. Thus, we compared changes in aesthetic judgments obtained four times in the experiment, which included initial judgments and changes in judgments correlated with the three types of background information.

Table 8 shows the pairwise comparisons of aesthetic judgments between the high and low art interest groups at different time points. This table shows at which time points the aesthetic judgments of tourists significantly differed.

### 3.5. The Correlation between Background and Art Interest and Aesthetic Judgment

The six factors of aesthetic judgment were tested by two-way repeated measures ANOVA. The results of Mauchly’s test of sphericity were all less than or equal to 0.001. This showed that all six measured factors of aesthetic judgment failed to satisfy the sphericity hypothesis and needed to be corrected for degrees of freedom; thus, the results of the Greenhouse–Geisser correction were used for all of them. The result of two-way repeated measures ANOVA is shown in Table 9.

The ANOVA results indicated that background information significantly affected all six measures of aesthetic judgment (*p* < 0.001) and showed no significant interaction between background information and art interest on all six measures of aesthetic judgment (*p* > 0.05). The background information factor had a significant effect on understanding (F = 18.875, *p* < 0.001), meaning (F = 17.158, *p* < 0.001), liking (F = 10.730, *p* < 0.001), interest (F = 8.043, *p* < 0.001), emotion (F = 14.008, *p* < 0.001), and ideas (F = 14.395, *p* < 0.001); that is, as background information continued to increase, tourists’ understanding, seeing meaning in the art, liking, interest, expression of emotion, and developing ideas about the blue calico as a tourist craft changed, as seen from the mean changes in Table 7. As background information continued to increase, aesthetic judgments increased in both the low and the high art interest groups. Thus, hypothesis 1 is supported.

The ANOVA results showed a significant effect of art interest on all six measures of aesthetic judgment (*p* < 0.001). Art interest is positively related to understanding (F = 42.390, *p* < 0.001), meaning (F = 41.436, *p* < 0.001), liking (F = 39.174, *p* < 0.001), interest (F = 44.858, *p* < 0.001), emotion (F = 47.237, *p* < 0.001), and ideas (F = 48.957, *p* < 0.001), all of which demonstrated significant effects. That is, a high level of art interest affected all six elements of aesthetic judgments, as evidenced by the means in Table 7, whereas the aesthetic judgments of the low art interest group were significantly lower than those of the high art interest group, regardless of the number of aesthetic judgments. Thus, hypothesis 2 is supported.

### 3.6. The Correlation between Different Levels of Art Interest and Aesthetic Judgment

**Figure 3:**
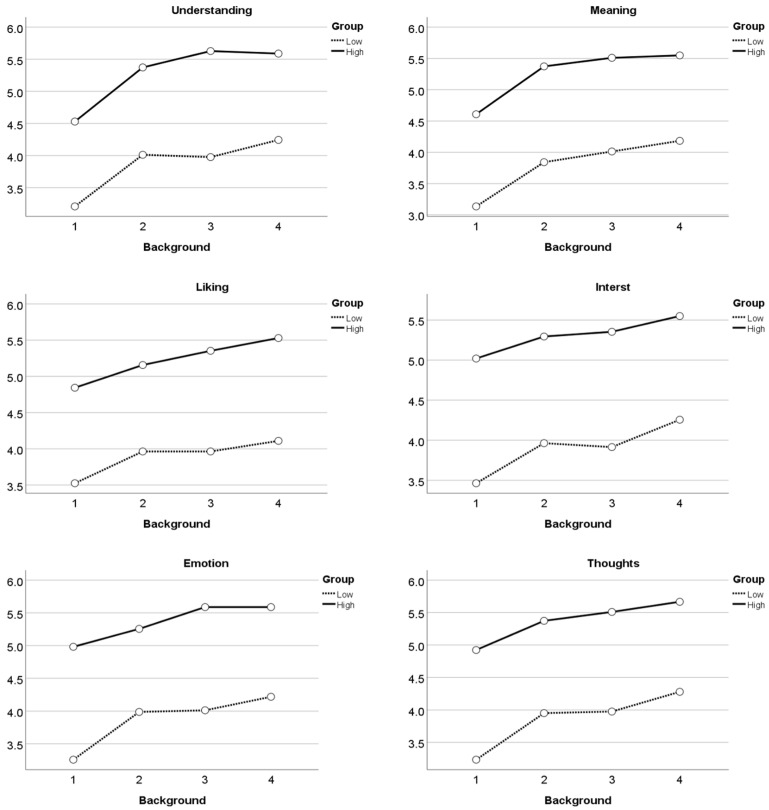
Interactive outline of background information and art interest.

Figure 3 shows the interaction contours of background information (T1, T2, T3, and T4) and art interest (high vs. low) for the six measured factors in aesthetic judgment.

Among the six factors of aesthetic judgment, the initial values of aesthetic judgment (T1) among the high art interest group were all higher than those of the low art interest group, indicating that participants with high art interest tended to have higher aesthetic appreciation and judgment ability. The six elements of aesthetic judgment of the high art interest group increased at T2, T3, and T4, while among the low art interest group, the six elements of aesthetic judgment increased at T2 and T4, the elements of understanding and interest decreased at T3, the element of liking remained unchanged, and the elements of emotion and idea rose only slightly. This indicates that the artist’s evaluation is positively related to aesthetic judgment of tourists with high art interest and is positively related to aesthetic judgment of tourists with low art interest only on the element of meaning. A comparison of T2, T3, and T4 indicated that the name had a positive correlation with aesthetic judgment for all participants, indicating that the name of the product is important in improving participants’ aesthetic judgment. Additionally, the production process is positively related to nearly all participants, especially those in the low art interest group. The video of the production process significantly increased their understanding, interest, liking, emotion, and thoughts about blue calico; however, there was no positive correlation between the aesthetic judgment of participants in the high art interest group and better understanding blue calico. Thus, our results support hypothesis 3, indicating that the aesthetic judgment of tourists with high and low interest in art is related to background information to different degrees.

## 4. Discussion

### 4.1. Findings

The main aim of this study was to evaluate the correlation between background information and aesthetics judgment of tourist crafts. In contrast to the aesthetic appreciation and judgment of artworks, the aesthetic judgment of tourist crafts and the mechanisms underlying their change are unclear. In this study, we assessed the aesthetic judgments of tourists with high and low art interest during repeated evaluations of tourist crafts. To facilitate tourists’ aesthetic appreciation of the crafts, we progressively provided three pieces of background information, including the name, the artist’s evaluation, and the production process. Three main findings were obtained from the study.

The first finding is that background information is positively related to the aesthetic judgment of tourist crafts, with the name having the greatest correlation, the production process the second, and the artist’s comments the least (and even negatively related with some aesthetic judgment factors). This indicates that for tourist crafts, the name of the work is very important for tourists’ aesthetic judgment, which can be effectively enhanced by a name with an auspicious meaning. The production process of tourist crafts is also an interesting aspect for tourists and an important feature that distinguishes handicrafts (which are perceived as more valuable) from machine-made products. In previous studies on paintings, the artist’s commentary has been shown to greatly enhance the aesthetic judgment of the viewer [39]. However, for this study, which focused on tourist crafts, this conclusion was not confirmed. Specifically, the artist’s evaluation has a positive correlation only on the element of meaning for tourists with low art interest. This may be the difference between the aesthetic judgment of high art and that of tourist crafts. People who appreciate high art value the evaluation of celebrities [39]; however, in the appreciation of tourist crafts, for people with a low art interest, the evaluation of celebrities is not very important.

The second finding is that art interest is positively related to the aesthetic judgment of tourists. The higher the art interest of the tourists, the higher their aesthetic judgment. We used the regression analysis of art interest as a continuous variable and used two-way repeated ANOVA after dichotomy of art interest. The results all support this finding. Using a continuous variable is more sensitive to different levels of variables. In addition, the dichotomy of continuous variables is helpful for in-depth analysis of the research results, and it was used in studies [71]. In fact, grouping tourists according to their art interest can provide useful suggestions for practical applications. By the same time, aesthetic judgments are heterogeneous. Tourists with high art interest have higher initial aesthetic judgments and aesthetic re-judgments than those with low art interest. This may be due not only to more experience in art appreciation, but also to higher levels of art knowledge. In addition, the aesthetic judgments of tourists with different art interests are related to background information to different degrees. The aesthetic judgement of tourists with high arts interest was less correlated with background information than that of tourists with low arts interest. A possible explanation for this might be that those with high art interests have formed their own aesthetic views and are less affected by new information.

The last finding is that the intrinsic psychological mechanisms of tourists’ aesthetic judgments are consistent with the model of aesthetic appreciation and aesthetic judgment proposed by Leder [38]. Domain-specific expertise, such as art interest, can predict tourists’ cognitive mastery, and aesthetic judgments are the result of the assessment of the cognitive mastery stage. The recognition of objects is divided into two types: explicit and invisible. Background information is attributed to explicit recognition. By increasing the explicit recognition of tourist crafts and the cognitive mastery of tourists, their aesthetic judgment can be effectively enhanced.

### 4.2. Theoretical Implications

First, our findings contribute to the literature on aesthetic judgment by demonstrating the correlation between tourists’ aesthetic judgments on tourist crafts and background information. To the best of our knowledge, the relationship between background information and aesthetic judgments of tourist crafts has not been empirically examined. Although aesthetic judgments are comprehensively discussed in the study of artwork [39,44,72,73,74], they have not received much attention in tourism studies. Thus, our study expands the scope of application of the concept of aesthetic judgment.

Second, the Vienna Art Interest and Art Knowledge (VAIAK) questionnaire is an internationally influential instrument for measuring art and aesthetics [46], but its applicability in Chinese contexts has not been studied. In this paper, we translate and fine-tune the scale in the Chinese context, verify its usability in the Chinese context through an empirical study, and provide a basic measurement tool for measuring art interest and knowledge in the Chinese context in the future.

Third, Leder et al.’s model of aesthetic appreciation and judgment as a classical aesthetic model has been discussed in many fields [38,75]; however, a gap remains in the field of tourism. In our study, the aesthetic judgment model was applied to the aesthetic study of tourism crafts, expanding the applicability of the model and verifying its applicability to tourism crafts.

### 4.3. Practical Implications

In addition to theoretical contributions, we also hope to contribute to the practice of creating tourist crafts. The study’s findings can provide policy recommendations for tourist craft manufacturers and sellers and contribute useful thinking to the heritage and development of tourist crafts, as well as intangible cultural heritage (ICH).

First, tourist craft stores should provide as much background information as possible to enhance tourists’ aesthetic judgment of their crafts, such as providing specific product names and symbolic meanings, showing documentaries, and displaying production processes and special titles. In addition, Esma [76] suggests that interactivity should be considered as a factor that affects artistic perception. The Hongyuantai Dyeing Workshop in Wuzhen, founded during the Song and Yuan dynasties, has been transformed into a tourist attraction in Wuzhen. The workshop shares a presentation of the entire procedure of the traditional process with visitors. If tourists can participate in the production process and take something away as a souvenir, it provides them with an unforgettable experience. Such measures could help those in the tourism industry market souvenirs to drive sales.

Second, in the context of the serious homogenization of tourism commodities and the lagging development of tourist crafts, our findings hope to promote the innovation and inheritance of tourist crafts and intangible cultural heritage (ICH). Tourism crafts are a form of commercialization of ICH. Enhancing the aesthetics of tourist crafts means enhancing the appreciation and understanding of ICH, which is beneficial to its preservation. Moreover, the diversification and perfection of tourism crafts can provide increasingly better choices for tourists, enhance the tourism experience with enjoyment, and meet the higher-level needs of tourists.

Third, with the normalization of COVID-19, an increasing number of countries are recovering in terms of tourism. Aesthetics are likely to be the focus of tourists’ attention. Tourist crafts have the opportunity to become new attractions for tourist destinations. In addition, the study of tourism aesthetics will help reconstruct the post-pandemic tourism development pattern and contribute to the emergence of new tourist destinations. Finally, with this empirical study of tourism aesthetics, we hope to promote the development of tourism crafts by studying their aesthetic attributes.

## 5. Conclusions

In summary, the most important finding to emerge from this study is that tourists’ impressions of the aesthetics of blue calicos were generally predicted by background information, especially among tourists who were less interested in high arts. Specifically, blue calicos reported to tourists as bearing names with an auspicious meaning predicted tourists’ assessments of them as more aesthetically pleasing. The explanations of the production process also predicted increased appreciation of blue calico aesthetics. This contrasted with artists’ commentaries, which were not significantly correlated with the increased aesthetic merit of blue calicos. Furthermore, this study revealed the heterogenicity of aesthetic judgment. The aesthetic judgments of people with different levels of art interest are diverse. In addition, this study has demonstrated that art interest plays a moderating role in the correlation between background information and aesthetic judgment. For people with different levels of art interest, their aesthetic judgments correlate with background information to varying degrees.

Our study used retrospective self-report questionnaires to confirm the positive roles of contextual information and art interest in aesthetic judgments at a psychological level. Nevertheless, it is not without limitations.

First, in art psychology, it has been demonstrated that the aesthetic appreciation of people’s activities predicts physiological reactions [77,78,79,80,81]. An EEG study found reduced gamma band activity in the left hemisphere of a viewer’s brain in association with increases in the subjective preference of artworks while viewing them [82]. Instruments, such as eye-movement experiments and EEG experiments, can be introduced in subsequent studies to draw conclusions at the physiological level. Combining these research tools to study the physiological changes in tourists correlated with aesthetics, e.g., vision (eye-tracking) and emotions (heart rate or skin conductance), and the nature and relationship between engagement and information processing (EEG) are very promising to advance the field of visitor experience and tourism aesthetics.

Second, in the context of cultural tourism integration, the aesthetic judgment of tourist crafts is an extremely important research direction. However, our study focused on only two influential factors—aesthetic judgment and art interest. In subsequent studies, additional factors, such as surrounding environment and introduction of sales, can be considered. In addition, it is possible to compare whether there are differences between aesthetic judgments in tourist and everyday life contexts and whether there are differences among the aesthetic judgments of tourists in different cultural contexts.

Third, as one of the few studies to examine the aesthetics of tourism crafts, our study uses a relatively simple research method, and the analysis results should be expanded. In the future studies, researchers can design more experiments for related studies, and produce more valuable and interesting results. Further studies can also focus on the specific behaviors of tourists, such as whether tourists will buy, the price they will accept, and other specific shopping behaviors. Finally, we sincerely hope that tourism aesthetics will attract increasing attention from researchers.

## Figures and Tables

**Table 1:** Sociodemographic information (*n* = 133).

Measure	Items	Frequency	Percentage
Gender	Men	84	63.2%
	Women	49	36.8%
Age	<20	23	17.3%
	20–25	66	49.6%
	26–30	38	28.6%
	>30	6	4.5%
Education level	Junior high school and below	9	6.8%
	High school/Technical school	18	13.5%
	Junior college	10	7.5%
	Bachelor	56	42.1%
	Master or above	40	30.1%
Occupation	Student	84	63.2%
	Blue-collar worker	5	3.7%
	Staff of enterprises & institutions	28	21.1%
	Other	16	12.0%
Monthly income (CNY)	<500	17	12.8%
	500–1000	16	12%
	1001–1500	15	11.3%
	1501–2000	18	13.5%
	2001–3000	20	15%
	3001–5000	12	9%
	5001–8000	13	9.8%
	8001–	11	8.3%
	>10,000	2	1.5%
	I don’t want to inform	9	6.8%

**Table 2:** Correlation analysis results.

	Gender	Age	Education Level	Occupation	Monthly Income
Art interest	0.956	0.648	0.453	0.947	0.939
Aesthetic judgment	0.510	0.437	0.107	0.312	0.903

**Table 3:** Results of exploratory factor analysis (EFA).

	Item	Factor			Communalities (Extraction)
		1	2	3	
Art interest	I enjoy talking about art with others	0.728			0.741
	I have many friends/acquaintances who are interested in art	0.580			0.562
	I come from a family that is interested in art	0.459			0.470
	I enjoyed attending art class in school	0.707			0.682
	During my everyday life I am interested in art	0.835			0.794
	During my everyday life I spontaneously notice art objects that I find fascinating	0.869			0.847
	During the tour, I am always looking for new artistic impressions and experiences	0.866			0.840
	During the tour, I like to visit artworks and art exhibitions	0.831			0.786
	During the tour, I like to participate in art creation activities	0.833			0.796
	During the tour, artistry is an important reason for my travel considerations	0.776			0.744
Behavioral frequency of art interest	How often do you visit art museums or art galleries on average?		0.795		0.663
	How often do you read books, magazines or catalogues about art?		0.829		0.797
	How often do you view images of artworks (picture books, internet, etc.)?		0.793		0.762
	How often do you visit events about art or art history (seminars, projects, festivals, etc.)?		0.798		0.739
Aesthetic judgment	Can you understand the patterns on the blue calicos?			0.861	0.813
	Do you have a clear understanding of the meaning of the patterns on the blue calicos?			0.852	0.817
	Do you like the patterns on the blue calicos?			0.821	0.784
	Are you interested in the patterns on the blue calicos?			0.828	0.799
	Do you have a clear idea of the emotions expressed in the patterns on the blue calicos?			0.849	0.877
	Did you get some ideas after looking at the patterns on the blue calicos?			0.805	0.791
Eigenvalue		6.512	3.387	5.206	
Explained variance (%)		32.561	16.937	26.028	

**Table 4:** Descriptive Statistics and Gender Differences in All Study Variables.

	Men (*n* = 84)	Women (*n* = 49)	Gender Effect		
	Mean ± SD	Mean ± SD	t	*p*	df
Art interest	161.75 ± 51.86	161.27 ± 41.43	0.056	0.956	131
Understanding	3.70 ± 1.90	3.73 ± 1.43	−0.111	0.912	122.768
Meaning	3.73 ± 1.92	3.65 ± 1.48	0.246	0.806	120.916
Liking	4.13 ± 1.68	3.86 ± 1.31	0.981	0.328	131
Interest	4.14 ± 1.78	3.92 ± 1.53	0.771	0.442	112.821
Emotion	3.99 ± 1.81	3.80 ± 1.47	0.632	0.529	131
Thoughts	4.01 ± 1.83	3.65 ± 1.55	1.151	0.252	131
Total aesthetic judgment	3.95 ± 1.66	3.77 ± 1.28	0.707	0.481	121.004

**Table 5:** Regression analysis summary for aesthetic judgment.

Variable	B	St. Error	β	t	*p*
(Constant)	−0.526	0.238		−2.206	0.029 **
Art interest	0.027	0.001	0.860	19.294	<0.001 ***

Note: ***: *p* < 0.001, **: *p* < 0.01.

**Table 6:** Cluster analysis results.

		Cluster 1 38.3% *n*: 51	Cluster 2 61.7% *n*: 82	*p*	(df)
Art interest	Q1	6	3	<0.001 ***	(1, 131)
	Q2	4	5	<0.001 ***	(1, 131)
	Q3	2	4	<0.001 ***	(1, 131)
	Q4	3	6	<0.001 ***	(1, 131)
	Q5	4	6	<0.001 ***	(1, 131)
	Q6	4	6	<0.001 ***	(1, 131)
	Q7	4	6	<0.001 ***	(1, 131)
	Q8	4	6	<0.001 ***	(1, 131)
	Q9	4	6	<0.001 ***	(1, 131)
	Q10	3	5	<0.001 ***	(1, 131)
Behavioral frequency of art interest	Q1	2	3	<0.001 ***	(1, 131)
	Q2	2	4	<0.001 ***	(1, 131)
	Q3	2	4	<0.001 ***	(1, 131)
	Q4	2	4	<0.001 ***	(1, 131)

Note: ***: *p* < 0.001.

**Table 7:** Descriptive analysis of aesthetic judgments.

Aesthetic Judgment	High Art Interest	Low Art Interest
	Mean ± SD	Mean ± SD
Understanding		
None (T1)	4.53 ± 1.68	3.21 ± 1.58
Product name (T2)	5.37 ± 1.25	4.01 ± 1.39
Artist’s Commentary (T3)	5.63 ± 1.31	3.98 ± 1.47
Production process (T4)	5.59 ± 1.28	4.24 ± 1.52
Meaning		
None (T1)	4.61 ± 1.65	3.13 ± 1.59
Product name (T2)	5.37 ± 1.41	3.84 ± 1.42
Artist’s Commentary (T3)	5.51 ± 1.36	4.01 ± 1.50
Production process (T4)	5.55 ± 1.42	4.18 ± 1.52
Liking		
None (T1)	4.84 ± 1.32	3.52 ± 1.48
Product name (T2)	5.16 ± 1.14	3.96 ± 1.44
Artist’s Commentary (T3)	5.35 ± 1.29	3.96 ± 1.41
Production process (T4)	5.53 ± 1.16	4.11 ± 1.43
Interest		
None (T1)	5.02 ± 1.44	3.46 ± 1.55
Product name (T2)	5.29 ± 1.20	3.96 ± 1.42
Artist’s Commentary (T3)	5.35 ± 1.31	3.91 ± 1.36
Production process (T4)	5.55 ± 1.21	4.26 ± 1.40
Emotion		
None (T1)	4.98 ± 1.36	3.26 ± 1.53
Product name (T2)	5.25 ± 1.47	3.99 ± 1.43
Artist’s Commentary (T3)	5.59 ± 1.17	4.01 ± 1.39
Production process (T4)	5.59 ± 1.33	4.22 ± 1.57
Thoughts		
None (T1)	4.92 ± 1.60	3.23 ± 1.49
Product name (T2)	5.37 ± 1.23	3.95 ± 1.42
Artist’s Commentary (T3)	5.51 ± 1.14	3.98 ± 1.44
Production process (T4)	5.67 ± 1.28	4.28 ± 1.47

**Table 8:** Pairwise comparisons.

	I	J	Mean Difference (I-J)	Std.Error	Sig. ^b^	95% CI for Difference ^b^	
						Lower Bound	Upper Bound
High art interest	T1	T2	−0.450 *	0.150	0.021 *	−0.855	−0.046
		T3	−0.658 *	0.164	0.001 **	−1.101	−0.215
		T4	−0.712 *	0.189	0.002 **	−1.225	−0.199
	T2	T1	0.450 *	0.150	0.021 *	0.046	0.855
		T3	−0.207	0.096	0.187	−0.466	0.052
		T4	−0.261	0.103	0.076	−0.539	0.017
	T3	T1	0.658 *	0.164	0.001 **	0.215	1.101
		T2	0.207	0.096	0.187	−0.052	0.466
		T4	−0.054	0.096	0.994	−0.314	0.206
	T4	T1	0.712 *	0.189	0.002 **	0.199	1.225
		T2	0.261	0.103	0.076	−0.017	0.539
		T3	0.054	0.096	0.994	−0.206	0.314
Low art interest	T1	T2	−0.796 *	0.152	<0.001 ***	−1.207	−0.386
		T3	−0.690 *	0.166	0.001 **	−1.139	−0.241
		T4	−1.023 *	0.192	<0.001 ***	−1.543	−0.503
	T2	T1	0.796 *	0.152	<0.001 ***	0.386	1.207
		T3	0.106	0.097	0.856	−0.156	0.369
		T4	−0.227	0.104	0.181	−0.509	0.055
	T3	T1	0.690 *	0.166	0.001 **	0.241	1.139
		T2	−0.106	0.097	0.856	−0.369	0.156
		T4	−0.333 *	0.097	0.006 **	−0.597	−0.070
	T4	T1	1.022 *	0.192	<0.001 ***	0.503	1.543
		T2	0.227 *	0.104	0.181	−0.055	0.509
		T3	0.333 *	0.097	0.006 **	0.070	0.597

Note: ***: *p* < 0.001, **: *p* < 0.01, *: *p* < 0.05. Based on estimated marginal means. *. The mean difference is significant at the 0.05 level. ^b^. Adjustment for multiple comparisons: Sidak.

**Table 9:** Results of two-way repeated measures ANOVA.

Variables	(df)	F	*p*	ηp ^2^
**Understanding**				
Background	(1, 131)	18.875	<0.001 ***	0.305
Art Interest	(1, 131)	42.390	<0.001 ***	0.244
Background × Art Interest	(1, 131)	1.615	0.189	0.036
**Meaning**				
Background	(1, 131)	17.158	<0.001 ***	0.285
Art Interest	(1, 131)	41.436	<0.001 ***	0.240
Background × Art Interest	(1, 131)	0.332	0.802	0.008
**Liking**				
Background	(1, 131)	10.730	<0.001 ***	0.200
Art Interest	(1, 131)	39.174	<0.001 ***	0.230
Background × Art Interest	(1, 131)	0.611	0.609	0.014
**Interest**				
Background	(1, 131)	8.043	<0.001 ***	0.158
Art Interest	(1, 131)	44.858	<0.001 ***	0.255
Background × Art Interest	(1, 131)	0.629	0.597	0.014
**Emotion**				
Background	(1, 131)	14.008	<0.001 ***	0.246
Art Interest	(1, 131)	47.237	<0.001 ***	0.265
Background × Art Interest	(1, 131)	1.499	0.218	0.034
**Thoughts**				
Background	(1, 131)	14.395	<0.001 ***	0.251
Art Interest	(1, 131)	48.957	<0.001 ***	0.272
Background × Art Interest	(1, 131)	0.588	0.624	0.013

Note: ***: *p* < 0.001.

## Data Availability

The data are openly available in FigShare at https://doi.org/10.6084/m9.figshare.17008363.v1 (accessed on 14 November 2021). Experimental materials and self-reported questionnaires are available from the corresponding author [K.L.].

## References

[1] Saarinen J. (2016). Cultural tourism and the role of crafts in Southern Africa: The case of craft markets in Windhoek, Namibia. Tour. Int. Interdiscip. J..

[2] Evans G. (1994). Tourism, the State of the Art.

[3] Torabian P., Arai S.M. (2016). Tourist perceptions of souvenir authenticity: An exploration of selective tourist blogs. Curr. Issues Tour..

[4] Swanson K.K., Timothy D.J. (2012). Souvenirs: Icons of meaning, commercialization and commoditization. Tour. Manag..

[5] Markwick M.C. (2001). Tourism and the development of handicraft production in the Maltese islands. Tour. Geogr..

[6] Graburn N.H. (1979). Ethnic and Tourist Arts: Cultural Expressions from the Fourth World.

[7] Jiang X. (2009). Design and Development of Tourist Souvenir of Jiangnan Blue Print. Master’s Thesis.

[8] Nantong Blue Calico Fabric Printing and Dyeing Technique. https://www.ihchina.cn/project_details/14296/.

[9] Knudsen D.C. (2015). Tourism, aesthetics, and touristic judgment. Tour. Rev. Int..

[10] Ackerman J.S. (2003). The photographic picturesque. Artibus Hist..

[11] Obasi N.T. (2015). Tourism aesthetics and values. J. Tour. Herit. Stud..

[12] Austin L.M. (2007). Aesthetic embarrassment: The reversion to the picturesque in nineteenth-century english tourism. ELH.

[13] MacKay K.J., Fesenmaier D.R. (1997). Pictorial element of destination in image formation. Ann. Tour. Res..

[14] Todd C. (2009). Philosophical. Issues in Tourism.

[15] Zaring J. (1977). The romantic face of wales. Ann. Assoc. Am. Geogr..

[16] Le D., Scott N., Becken S., Connolly R.M. (2019). Tourists’ aesthetic assessment of environmental changes, linking conservation planning to sustainable tourism development. J. Sustain. Tour..

[17] Schirpke U., Timmermann F., Tappeiner U., Tasser E. (2016). Cultural ecosystem services of mountain regions: Modelling the aesthetic value. Ecol. Indic..

[18] Kirillova K., Fu X., Lehto X., Cai L. (2014). What makes a destination beautiful? Dimensions of tourist aesthetic judgment. Tour. Manag..

[19] Zhuang M., Zhang J., Xiao X., Qiu M., Lu Y., Zhang H., Tseng T., Zuo L., Hu M. (2020). How destination music affects tourists’ behaviors: Travel with music in Lijiang, China. Asia Pac. J. Tour. Res..

[20] Marder B., Erz A., Angell R., Plangger K. (2021). The role of photograph aesthetics on online review sites: Effects of management- versus traveler-generated photos on tourists’ decision making. J. Travel Res..

[21] Pajin D. (1997). Environmental aesthetics and chinese gardens. Dialogue Univers..

[22] Zuckert R. (2009). Sculpture and touch: Herder’s aesthetics of sculpture. J. Aesthet. Art Crit..

[23] He M., He X., Zheng Z., Deng J., Wu C. (2022). The impact of action observation and motor imagery on the aesthetic preference of Chinese calligraphy. PsyCh J..

[24] Scruton R. (2021). The Aesthetics of Architecture.

[25] Schifferstein H.N.J., Kudrowitz B.M., Breuer C. (2020). Food perception and aesthetics-Linking sensory science to culinary practice. J. Culin. Sci. Technol..

[26] Hagen L. (2021). Pretty healthy food: How and when aesthetics enhance perceived healthiness. J. Mark..

[27] Haller C., Hess-Misslin I., Mereaux J. (2021). Aesthetics and conviviality as key factors in a successful wine tourism experience. Int. J. Wine Bus. Res..

[28] Krasovska O., Miskova N., Veremchuk A. (2020). Professional training of future preschool teachers in the field of artistic and aesthetic education by means of contextual learning technologies. Behav. Sci..

[29] Silvia P.J., Rodriguez R.M., Cotter K.N., Christensen A.P. (2021). Aesthetic preference for glossy materials: An attempted replication and extension. Behav. Sci..

[30] Mari E., Quaglieri A., Lausi G., Boccia M., Pizzo A., Baldi M., Barchielli B., Burrai J., Piccardi L., Giannini A.M. (2021). Fostering the aesthetic pleasure: The effect of verbal description on aesthetic appreciation of ambiguous and unambiguous artworks. Behav. Sci..

[31] Xu X.E., Le T.H., Kwek A., Wang Y. (2022). Exploring cultural tourist towns: Does authenticity matter?. Tour. Manag. Perspect..

[32] Mastandrea S., Fagioli S., Biasi V. (2019). Art and psychological well-being: Linking the brain to the aesthetic emotion. Front. Psychol..

[33] He Z., Wu L., Li X.R. (2018). When art meets tech: The role of augmented reality in enhancing museum experiences and purchase intentions. Tour. Manag..

[34] Urry J. (2000). Mobile sociology. Br. J. Sociol..

[35] Sun S., Law R., Zhang M. (2020). An updated review of tourism-related experimental design articles. Asia Pac. J. Tour. Res..

[36] Pan H. (2016). Analysis of tourism beauty appreciation based on living aesthetics: From sightseeing to leisure. Tour. Trib..

[37] Ray J., Pine T.L.H.Q. (2005). Tourism and Hotel Development in China: From Political to Economic Success.

[38] Leder H., Belke B., Oeberst A., Augustin D. (2004). A model of aesthetic appreciation and aesthetic judgments. Br. J. Psychol..

[39] Park S.A., Yun K., Jeong J. (2015). Reappraising abstract paintings after exposure to background information. PLoS ONE.

[40] Dawson G., Toth K., Abbott R., Osterling J., Munson J., Estes A., Liaw J. (2004). Early social attention impairments in autism: Social orienting, joint attention, and attention to distress. Dev. Psychol..

[41] Kveraga K., Boshyan J., Bar M. (2007). Magnocellular projections as the trigger of top-down facilitation in recognition. J. Neurosci..

[42] Silvia P.J. (2013). Interested experts, confused novices: Art expertise and the knowledge emotions. Empir. Stud. Arts.

[43] Hume D. (1757). Four Dissertations: I. The Natural History of Religion. II. of the Passions. III. of Tragedy. IV. of the Standard of Taste.

[44] Leder H., Carbon C., Ripsas A. (2006). Entitling art: Influence of title information on understanding and appreciation of paintings. Acta Psychol..

[45] Pelowski M., Markey P.S., Lauring J.O., Leder H. (2016). Visualizing the impact of art: An update and comparison of current psychological models of art experience. Front. Hum. Neurosci..

[46] Specker E., Forster M., Brinkmann H., Boddy J., Pelowski M., Rosenberg R., Leder H. (2020). The Vienna Art Interest and Art Knowledge Questionnaire (VAIAK): A unified and validated measure of art interest and art knowledge. Psychol. Aesthet. Creat. Arts.

[47] Featherstone M. (2007). Consumer Culture and Postmodernism.

[48] Wakefield K.L., Blodgett J.G. (1996). The effect of the servicescape on customers’ behavioral intentions in leisure service settings. J. Serv. Mark..

[49] Ha J., Jang S.S. (2012). The effects of dining atmospherics on behavioral intentions through quality perception. J. Serv. Mark..

[50] Liu Y., Jang S.S. (2009). The effects of dining atmospherics: An extended Mehrabian–Russell model. Int. J. Hosp. Manag..

[51] Tractinsky N., Katz A.S., Ikar D. (2000). What is beautiful is usable. Interact. Comput..

[52] Kurosu M.K.K. Apparent usability vs. inherent usability: Experimental analysis on the determinants of the apparent usability. Proceedings of the CHI’95.

[53] Tuch A.N., Bargas-Avila J.A., Opwis K. (2010). Symmetry and aesthetics in website design: It’s a man’ s business. Comput. Hum. Behav..

[54] Thüring M., Mahlke S. (2007). Usability, aesthetics and emotions in human–technology interaction. Int. J. Psychol..

[55] De Angeli A.S.A.H. Interaction, usability and aesthetics: What influences users’ preferences?. Proceedings of the 6th Conference on Designing Interactive Systems.

[56] Quinn J.M., Tran T.Q. (2010). Attractive phones don’t have to work better. Proceedings of the 28th International Conference on Human Factors in Computing Systems—CHI’10.

[57] van Schaik P., Ling J. (2011). An integrated model of interaction experience for information retrieval in a Web-based encyclopaedia. Interact. Comput..

[58] van Schaik P., Ling J. (2008). Modelling user experience with web sites: Usability, hedonic value, beauty and goodness. Interact. Comput..

[59] Schellekens E.A.P.G. (2011). The Aesthetic Mind: Philosophy and Psychology.

[60] Pikkemaat B., Weiermair K. (2003). The Aesthetic (Design) Orientated Customer in Tourism-Implications for Product Development.

[61] Cohen J. (1977). Statistical Power Analysis for the Behavioral Sciences.

[62] Leenaars C.H., Zant J.C., Aussems A., Faatz V., Snackers D., Kalsbeek A. (2016). The Leeds food preference questionnaire after mild sleep restriction—A small feasibility study. Physiol. Behav..

[63] Jiangsu Intangible Cultural Heritage. http://www.jsfybh.com.

[64] Live China. http://livechina.cctv.com.

[65] Child D. (2006). The Essentials of Factor Analysis.

[66] Maskey R., Fei J., Nguyen H. (2018). Use of exploratory factor analysis in maritime research. Asian J. Shipp. Logist..

[67] Arifin W.N. (2018). A web-based sample size calculator for reliability studies. Educ. Med. J..

[68] Williams B., Onsman A., Brown T. (2010). Exploratory factor analysis: A five-step guide for novices. Australas. J. Paramed..

[69] Kim J., Mueller C.W. (1978). Factor Analysis: Statistical Methods and Practical Issues.

[70] Mirzaei N., Dehdari T., Taghdisi M.H., Zare N. (2019). Development of an instrument based on the theory of planned behavior variables to measure factors influencing Iranian adults’ intention to quit waterpipe tobacco smoking. Psychol. Res. Behav. Manag..

[71] Bloch P.H., Brunel F.F., Arnold T.J. (2003). Individual differences in the centrality of visual product aesthetics: Concept and measurement. J. Consum. Res..

[72] Szubielska M., Imbir K., Szymańska A. (2021). The influence of the physical context and knowledge of artworks on the aesthetic experience of interactive installations. Curr. Psychol..

[73] Calvo-Merino B., Jola C., Glaser D.E., Haggard P. (2008). Towards a sensorimotor aesthetics of performing art. Conscious. Cogn..

[74] Belke B., Leder H., Augustin M.D. (2006). Mastering style-Effects of explicit style-related information, art knowledge and affective state on appreciation of abstract paintings. Psychol. Sci..

[75] Leder H., Nadal M. (2014). Ten years of a model of aesthetic appreciation and aesthetic judgments: The aesthetic episode-Developments and challenges in empirical aesthetics. Br. J. Psychol..

[76] Savaş E.B., Verwijmeren T., van Lier R. (2021). Aesthetic experience and creativity in interactive art. Art Percept..

[77] Ticini L.F. (2017). The role of the orbitofrontal and dorsolateral prefrontal cortices in aesthetic preference for art. Behav. Sci..

[78] Jacobs A.M. (2015). Neurocognitive poetics: Methods and models for investigating the neuronal and cognitive-affective bases of literature reception. Front. Hum. Neurosci..

[79] Scott N., Le D., Becken S., Connolly R.M. (2020). Measuring perceived beauty of the Great Barrier Reef using eye-tracking technology. Curr. Issues Tour..

[80] Liu J., Lughofer E., Zeng X. (2017). Toward model building for visual aesthetic perception. Comput. Intell. Neurosc..

[81] Boccia M., Barbetti S., Piccardi L., Guariglia C., Ferlazzo F., Giannini A.M., Zaidel D.W. (2016). Where does brain neural activation in aesthetic responses to visual art occur? Meta-analytic evidence from neuroimaging studies. Neurosci. Biobehav. Rev..

[82] Lengger P.G., Fischmeister F.P.S., Leder H., Bauer H. (2007). Functional neuroanatomy of the perception of modern art: A DC–EEG study on the influence of stylistic information on aesthetic experience. Brain Res..

